# Pharmacological treatment of perinatal depression in women with ADHD

**DOI:** 10.1192/j.eurpsy.2026.10310

**Published:** 2026-07-31

**Authors:** O. Kilic

**Affiliations:** Department of Neurosciences, Bezmialem Vakif University, Institute of Health Sciences, Istanbul, Türkiye

## Abstract

**Abstract:**

Perinatal depression affects 20–28% of women globally, with prevalence rates significantly higher among those with comorbid Attention-Deficit/Hyperactivity Disorder (ADHD), with self-reported symptoms exceeding 50% in clinical samples. Hormonal fluctuations during pregnancy and the postpartum period can exacerbate both ADHD symptoms and depressive episodes, leading to increased symptom severity. ADHD is linked to higher depression burden and greater risk of antidepressant treatment resistance in general. Despite this elevated risk, pharmacological guidelines specifically for treating perinatal depression comorbid with ADHD are lacking.

In the absence of specific pharmacological guidelines, current evidence on the pharmacological treatment of perinatal depression in general has shown that selective serotonin reuptake inhibitors (SSRIs)—specifically sertraline- are the first-line pharmacological treatments due to their favourable safety profiles during pregnancy and lactation. In severe or treatment-resistant depression, emerging neuroactive steroids like brexanolone and zuranolone offer rapid symptom relief, though long-term data remain limited.

Clinicians must weigh the small absolute risks of antidepressant use—such as preterm birth and lower APGAR scores—against the significant risks of untreated depression. Management requires individualised risk-benefit analysis. Furthermore, pregnancy-related pharmacokinetic changes may necessitate therapeutic drug monitoring and dose adjustments to maintain therapeutic efficacy. Clinically, this supports closer monitoring and a low threshold for adjusting or augmenting antidepressant treatment.

Pharmacotherapy for comorbid ADHD and perinatal depression involves greater uncertainty. The safety and efficacy of continuing stimulant or non-stimulant ADHD medications during pregnancy are under-researched and not well established. The safety of the adjustment of stimulant dosage during pregnancy has not been proven. Consequently, the concurrent use of ADHD medications and antidepressants requires scrutiny and a multidisciplinary, individualised approach.

Regarding breastfeeding, sertraline and paroxetine are considered compatible; however, due to the indeterminate safety profile of ADHD medications in lactation, decisions regarding their continuation must be made on a case-by-case basis.

Future research is critically needed to establish the safety of combined antidepressant and ADHD medication regimens to improve outcomes for this high-risk population.

**Disclosure of Interest:**

None Declared

